# Supplementary material for: Antibacterial, Antioxidation, UV-Blocking, and Biodegradable Soy Protein Isolate Food Packaging Film with Mangosteen Peel Extract and ZnO Nanoparticles
Source: Nanomaterials (Basel). 2021 Dec 8;11(12):3337. doi: 10.3390/nano11123337 (PMC8707035; doi:10.3390/nano11123337)
Supplement: Supplementary file 1 [file nanomaterials-11-03337-s001.zip › nanomaterials-1456430-supplementary.pdf]

Supplementary Materials

Antibacterial, Antioxidation, UV-Blocking, and Biodegradable Soy Protein Isolate Food Packaging Film with Mangosteen Peel Extract and ZnO Nanoparticles

Xi Huang [1], Xin Zhou [1], Qingyin Dai [1] and Zhiyong Qin [1,2,*]

[1] School of Resources, Environment, and Materials, Guangxi University, Nanning 530000, China; 2015391083@st.gxu.edu.cn (X.H.); 13768371312@163.com (X.Z.); wdmzsdqf@gmail.com (Q.D.)
[2] MOE Key Laboratory of New Processing Technology for Non-Ferrous Metals and Materials; Nanning 530000, China
* Correspondence: q_zhiyong@163.com; Tel.: +86-182-7710-5246

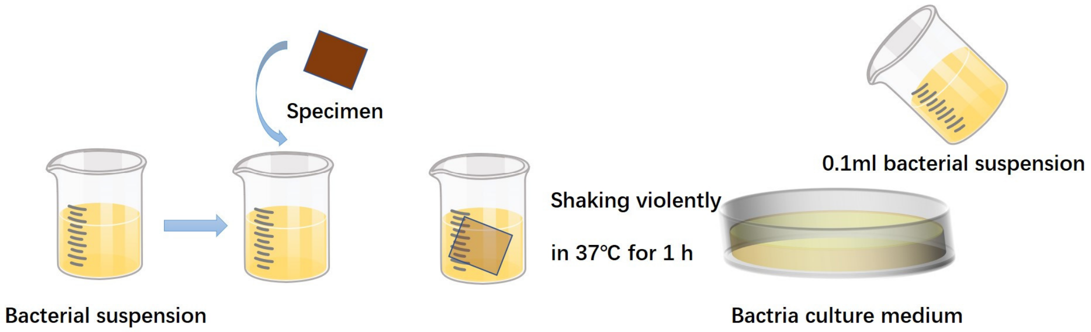

Figure S1. Schematic illustration of the method of antibacterial experiment.

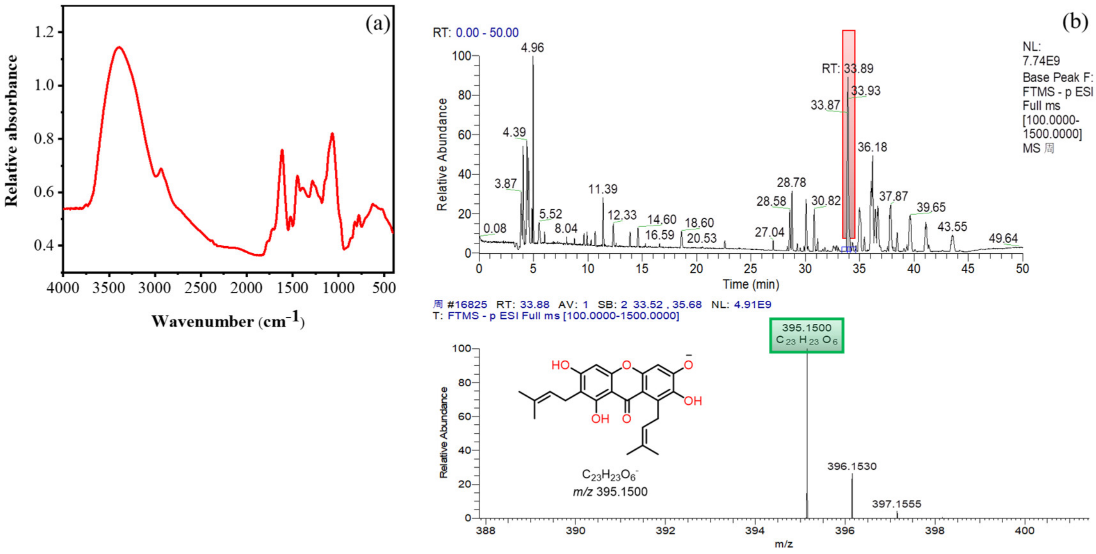

Figure S2. Absorption spectrum of MPE (**a**) and analysis of the structure of MPE by HPLC (**b**). As in the base peak ion outflow diagram, the presence of the suspected compounds in the MPE is shown at different moments. The time of 33.89 corresponds to the molecular weight of the suspected compound of 395.1500.

the molecular formula of the compound is $C_{23}H_{23}O_6$, which corresponds to the main component of MPE, γ-mangosteen, according to the primary spectrum and the resolution results.

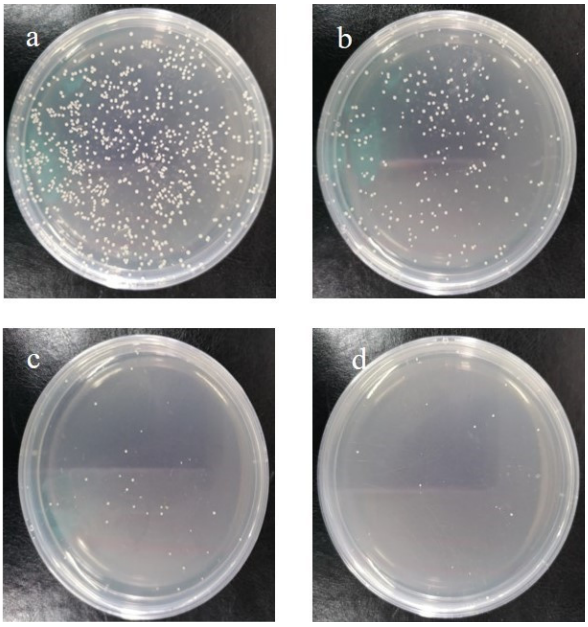

Figure S3. Antibacterial effect of SPI/MPE/ZnO films against *E. coli*. SPI control(**a**); SPI/MPE/ZnO$^{1\%}$(**b**); SPI/MPE/ZnO$^{3\%}$(**c**) and SPI/MPE/ZnO$^{5\%}$(**d**).

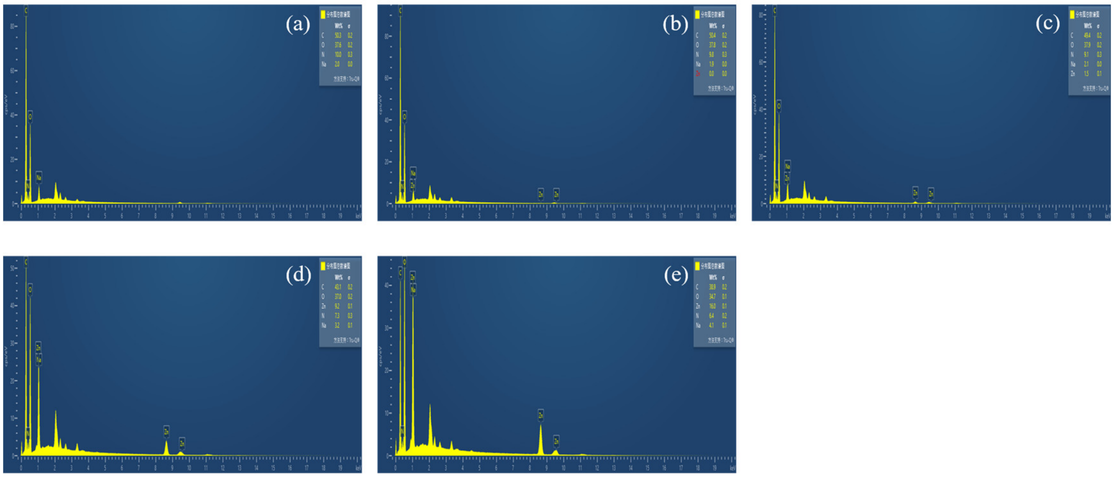

Figure S4. EDX spectra of SPI films for SPI control (**a**), SPI/MPE (**b**), SPI/MPE/ZnO$^{1\%}$(**c**), SPI/MPE/ZnO$^{3\%}$(**d**) and SPI/MPE/ZnO$^{5\%}$(**e**) film, respectively.

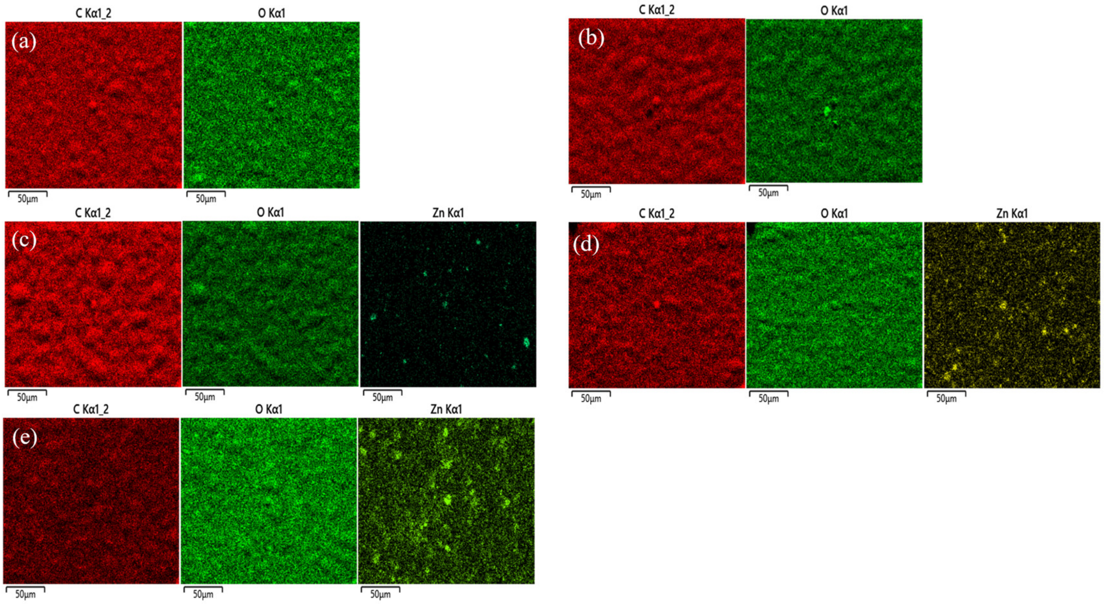

Figure S5. The elemental mapping analysis (MAP) for SPI control (**a**), SPI/MPE (**b**), SPI/MPE/ZnO$^{1\%}$(**c**), SPI/MPE/ZnO$^{3\%}$(**d**) and SPI/MPE/ZnO$^{5\%}$ (**e**) film, respectively.

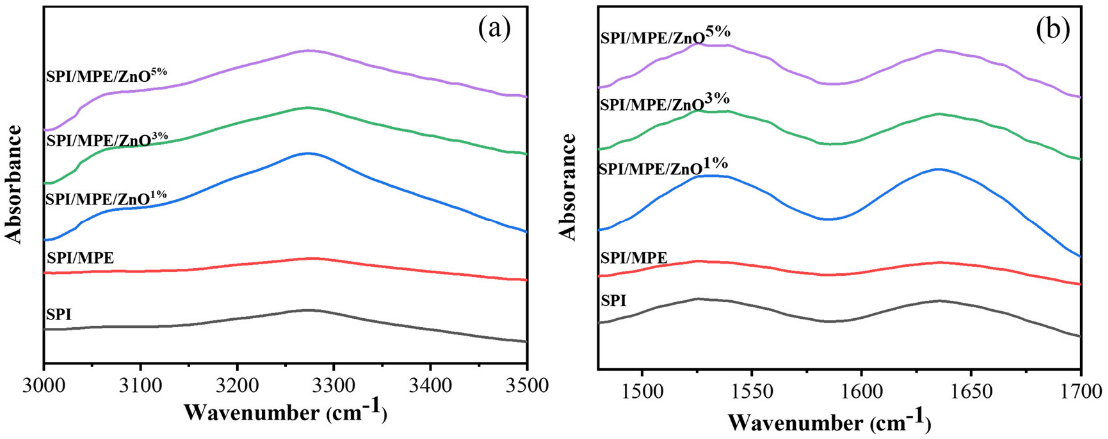

Figure S6. ATR-FTIR spectra of SPI films for amide-A (A) and amide I (B).

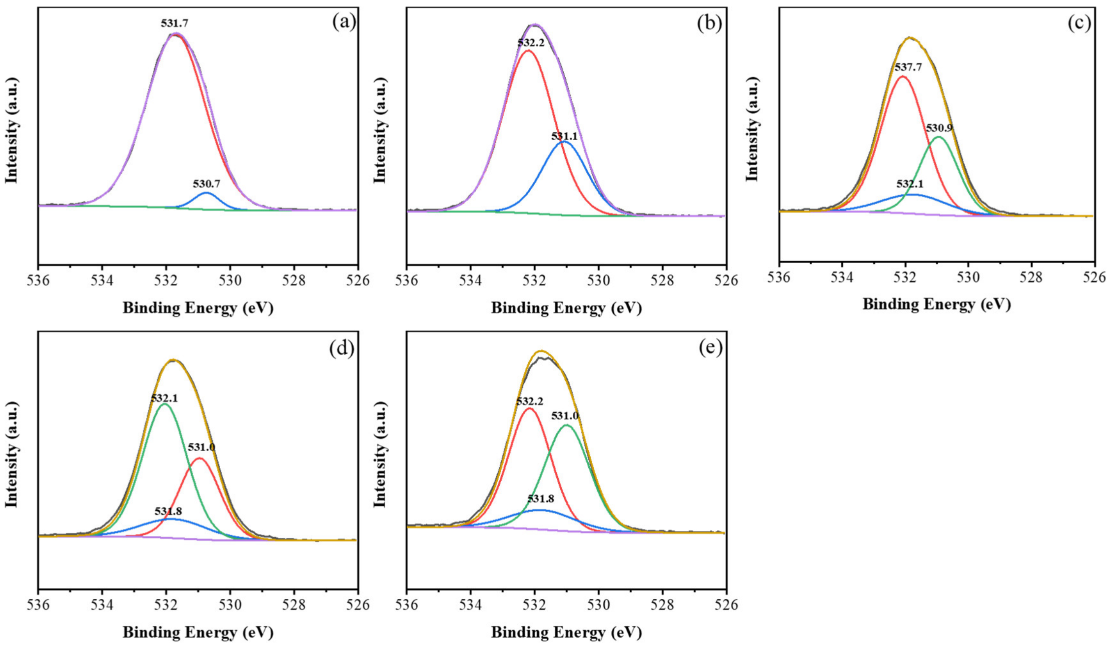

Figure S7. XPS of O 1s features of SPI films for SPI control (**a**), SPI/MPE (**b**), SPI/MPE/ZnO$^{1\%}$ (**c**), SPI/MPE/ZnO$^{3\%}$ (**d**) and SPI/MPE/ZnO$^{5\%}$ (**e**) film, respectively.

Table S1. Experimental formulations of the SPI films.

Films	5% SPI (g) [a]	0.5% CMC (g) [b]	Glycerol (g) [c]	0.5% ZnO (g)[d]	MPE (g)[e]
SPI	25	5	0.375	0	0
SPI/MPE	25	5	0.375	0	0. 125
SPI/MPE/ZnO[1%]	25	5	0.375	2.5	0.125
SPI/MPE/ZnO[3%]	25	5	0.375	7.5	0.125
SPI/MPE/ZnO[5%]	25	5	0.375	12.5	0.125

[a] 5 wt% solution.

[b] 0.5 wt% solution ,2 wt% based on dry weight of SPI.

[c] 30 wt% on a dry weight basis of SPI.

[d] 0.5 wt% solution, 0, 1, 3, 5 wt% on a dry weight basis of SPI.

[e] 10 wt% on a dry weight basis of SPI.

Table S2. Colour and opacity of SPI films as affected by MPE and ZnO NPs incorporation.

Films	L*	a*	b*	ΔE*	Opacity values
SPI	86.34 ± 0.19[a]	0.91 ± 0.26[e]	20.92 ± 0.16[e]	5.44 ± 0.20[d]	1.07 ± 0.20[d]
SPI/MPE	74.50 ± 0.31[b]	8.49 ± 0.01[d]	38.02 ± 0.21[d]	27.60 ± 0.32[c]	1.47 ± 0.27[d]
SPI/MPE/ZnO[1%]	65.02 ± 0.02[c]	15.54 ± 0.06[a]	50.16 ± 0.26[a]	44.44 ± 0.19[a]	2.24 ± 0.02[c]
SPI/MPE/ZnO[3%]	62.28 ± 0.12[d]	14.23 ± 0.02[b]	48.59 ± 0.3[b]	44.27 ± 0.26[a]	3.33 ± 0.64[b]
SPI/MPE/ZnO[5%]	62.20 ± 0.03[d]	13.24 ± 0.08[c]	46.86 ± 0.22[c]	42.73 ± 0.14[b]	6.12 ± 0.66[a]

*All data were expressed as mean ± standard deviation (SD). Different letters indicated significant difference (p≤0.05).

Table S3. Antibacterial properties of SPI films incorporated with ZnO NPs.

Films<br>Bacterial colony	SPI	SPI/ZnO[1%]	SPI/ZnO[3%]	SPI/ZnO[5%]
E. coli	845 ± 9.90[a]	539.5 ± 31.82[b]	78.50 ± 10.61[c]	76.00 ± 11.31[c]
Bactericidal rate (%)	0	36.83	90.81	91.10
S. aureus	1143.00 ± 53.74[a]	105.50 ± 2.12[b]	24.00 ± 0.00[c]	12.50 ± 2.12[c]
Bactericidal rate (%)	0	90.77	97.90	98.91

All data were expressed as mean ± standard deviation (SD). Different letters indicated significant difference (p≤0.05).

Table S4. Roughness of SPI films.

Films Roughness	SPI	SPI/MPE	SPI/MPE/ZnO1%	SPI/MPE/ZnO3%	SPI/MPE/ZnO5%
Average roughness, Ra (nm)	11.663	6.765	9.032	9.238	19.729
Root mean square roughness, Rq (nm)	15.844	9.663	11.683	12.326	27.059

Where Ra represents the arithmetic mean value of the surface height deviation determined from the average plane; Rq is the root mean square of the height deviation derived from the average image data plane.

Table S5 Mass loss calculated from thermal parameters of the TGA curve.

Films	LM_1 (%)	Td_{max1}(℃)	LM_2 (%)	Td_{max2} (℃)	RM (%)
SPI control	11.13	99.74	66.06	306.37	22.39
SPI/MPE	9.63	100.42	67.22	306.86	23.15
SPI/MPE/ZnO1%	10.98	79.06	61.78	300.96	27.25
SPI/MPE/ZnO3%	12.38	67.44	59.76	302.53	27.86
SPI/MPE/ZnO5%	14.15	72.71	56.23	269.14	29.63

LM_1=loss of mass in the first event (dehydration), LM_2=second event (decomposition), RM=residual mass at 650 °C.

Table S6 Relative amounts (%) of carbon (C 1s) on the surfaces of SPI films.

Films	C1 (%)	C2 (%)	C3 (%)
SPI control	66.40	19.81	13.79
SPI/MPE	58.08	26.00	15.92
SPI/MPE/ZnO1%	58.03	26.35	15.62
SPI/MPE/ZnO3%	60.04	24.86	15.10
SPI/MPE/ZnO5%	63.43	22.22	14.35
